# Aberrant mediastinal basal pulmonary artery encountered at anatomical lung resection: A case report and review of the literature

**DOI:** 10.1016/j.ijscr.2024.109394

**Published:** 2024-02-21

**Authors:** Hideki Itano, Masayuki Yamaji, Masashi Yoshihara

**Affiliations:** a Department of Thoracic Surgery, Daiyu-kai General Hospital, Ichinomiya-shi, Aichi, Japan; b Department of Thoracic Surgery, Otokoyama Hospital, Yawata-shi, Kyoto, Japan

**Keywords:** Pulmonary artery variation, Mediastinal basal pulmonary artery, Arteria Praebronchialis, 3D-CECT angiography, Video-assisted thoracoscopic surgery, Case report, Literature review

## Abstract

**Introduction:**

Abnormal branching of the pulmonary artery is often encountered in anatomical lung resection, which can potentially result in accidental vessel injury with life-threatening bleeding or extra lung resection. The mediastinal basal pulmonary artery (Arteria Praebronchialis, AP) is a very rare but potentially critical variant.

**Presentation of case:**

We present the case of a patient with lung cancer accompanied by the left basal segmental pulmonary artery, independent A^8a+9^, which was liable to be injured during lower lobectomy with poor interlobar fissure development. This variation was preoperatively recognized using three-dimensional contrast-enhanced computed tomography (3D-CECT) angiography, and vessel injury was avoided.

**Discussion and literature review:**

3D-CECT angiography was effective in identifying this rare but potentially critical variation, and it is desirable to perform it routinely before anatomical lung resection. A review of 31 AP cases revealed that the branching pattern of AP was independent (15 patients, 48 %) and common trunk type (16 patients, 52 %), one half for each. Mediastinal branching of the lingular artery was more frequent among the reported AP cases (71 %) than in general reports.

**Conclusion:**

When mediastinal branches of left pulmonary artery are encountered, the possibility that it is AP should be always taken into account.

## Highlights

• A lung cancer patient with AP, A^8a+9^, underwent safe thoracoscopic left lower lobectomy.
• 3D-CECT angiography is effective in identifying this rare but potentially critical variation.
• Branching pattern of 31 AP cases comprised independent and common trunk types, with one-half each.
• AP cases were more frequently accompanied by a mediastinal lingular artery (71 %) than generally reported.

## 1 Introduction

Abnormal branching of the pulmonary artery is often encountered during anatomical lung resections. A mediastinal basal pulmonary artery on the left side, also known as Arteria Praebronchialis (AP), is a very rare anatomical variation (0.05 %) [1], but can be potentially critical by mistaking it for the mediastinal lingular artery to result in accidental extra-lung resection or severe bleeding. Herein, we present a case of AP detected on preoperative 3D-CECT angiography that underwent safe left lower lobectomy for lung cancer. In addition, literature on 31 AP cases was reviewed.

## 2 Presentation of case

**Fig. 1:**
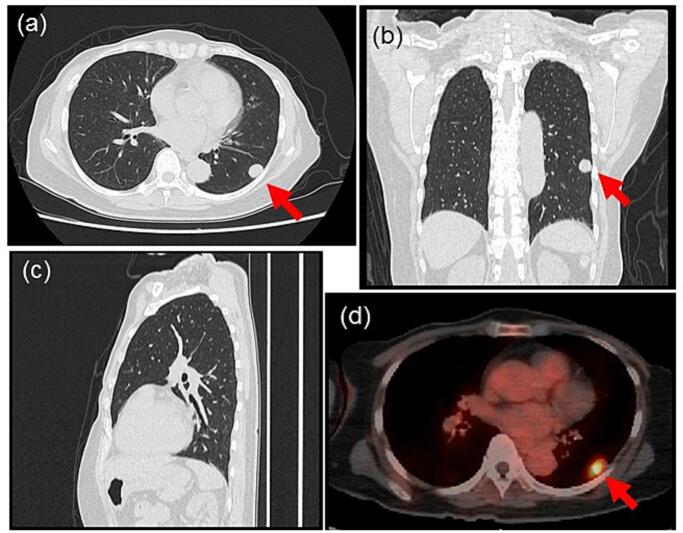
Preoperative chest computed tomography (CT) and FDG-PET/CT images. (a) CT, axial section image; (b) CT, coronal section image: primary lung adenocarcinoma in the left lower lobe S^9^a, 18 × 12 × 16 mm (red arrow); (c) CT, sagittal section image showing a poorly developed interlobar fissure of the left lung. (d) FDG-PET/CT: FDG uptake into the cancer is significant (SUVmax: 7.44) (red arrow). (For interpretation of the references to colour in this figure legend, the reader is referred to the web version of this article.)

**Fig. 2:**
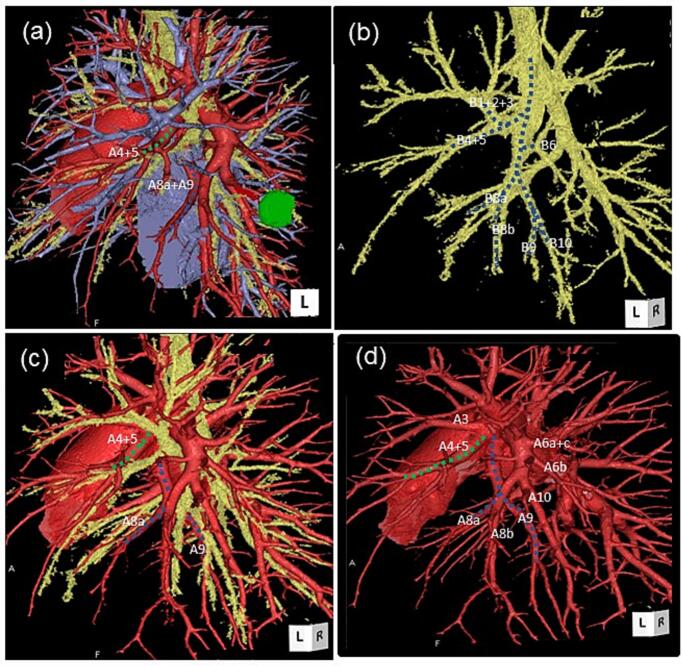
3D-CECT angiography images. (a) Combination 3D image of the pulmonary vessels and bronchial tree: green dotted line: A^4+5^, blue dotted line: aberrant mediastinal basal artery A^8^^a^^+9^. Green mass: S^9^a lung cancer. (b) 3D image of the bronchial tree; the left bronchial tree is depicted with a blue dotted line. (c) Combination 3D image of the pulmonary artery and bronchial tree: green dotted line: A^4+5^, blue dotted line: aberrant mediastinal basal artery A^8^^a^^+9^. By utilizing a combination of 3D images of the accompanying pulmonary artery and bronchial tree, accurate identification of AP, A^8^^a^^+9^^,^ becomes easier. (d) 3D image of the pulmonary artery: green dotted line: independent A^4+5^; blue dotted line: independent AP, A^8^^a+^^9^. (For interpretation of the references to colour in this figure legend, the reader is referred to the web version of this article.)

**Fig. 3:**
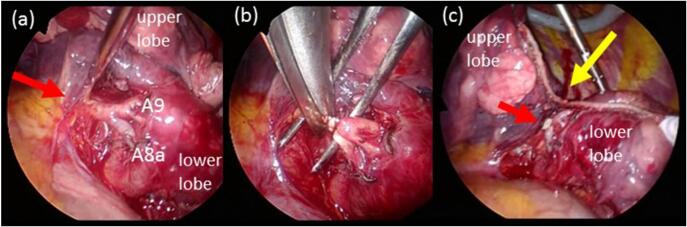
Operative findings. (a) The aberrant mediastinal basal artery (AP), A^8^a + A^9^ (red arrow) enters the lower lobe from the anteromedial aspect. (b) The division of aberrant A^8a+9^. (c) Creation of the interlobar fissure (yellow arrow) and stump of A^8a+9^ (red arrow). (For interpretation of the references to colour in this figure legend, the reader is referred to the web version of this article.)

A 69-year-old woman with a history of bilateral breast cancer surgery was diagnosed with primary lung adenocarcinoma in the left lower lobe S^9^a, 18 × 12 × 16 mm in size, which was asymptomatic, identified by follow-up CT scan, and histologically diagnosed by CT-guided needle biopsy. The disease was classified as c-T1bN0M0 stage IA 2, according to the Union for International Cancer Control (UICC) classification (seventh edition), following fluorodeoxyglucose (FDG)-positron emission tomography (PET)/CT (Fig. 1) and brain magnetic resonance imaging. Preoperative 3D-CECT angiography clearly detected an anatomical variant of the left basal segmental pulmonary artery A^8a+9^, which branches independently from the proximal portion of the left main pulmonary trunk medio-distally adjacent to the independent mediastinal A^4+5^, descends behind both the upper pulmonary vein and the upper lobe bronchus, and flows into the basal segments S^8^a and S^9^ just lateral to the inferior pulmonary vein on the anteromedial aspect of the lower lobe. 3D-CECT angiography also demonstrated an independently branching mediastinal lingular artery (A^4+5^) without interlobar branches (Fig. 2). Extremely poor development of the interlobar fissure of the left lung was also observed on CT (Fig. 1c). There were no anomalies of the bronchi or pulmonary veins. Video-assisted thoracoscopic left lower lobectomy and lymph node dissection were performed for lung cancer without injuring the AP, A^8a+9^. After identifying the aberrant A^8a+9^ branch entering the lower lobe from the anteromedial aspect (Fig. 3a), the pulmonary artery trunk was posteriorly dissected, and the A^6^ branch was dissected and divided first. A poorly developed interlobar fissure was plicated by dividing the lung parenchyma step-by-step along the lateral surface of the pulmonary artery trunk in the direction from the posterior to anterior using a stapling device. Just before separation of the most anterior portion of the interlobar fissure, the aberrant A^8a+9^ segmental pulmonary artery branch was ligated and divided to avoid injury (Fig. 3b, c; Video 1). Following division of the main trunk of the pulmonary artery (A^8a^+^10^) to the lower lobe in the interlobar fissure by stapling, the lower lobe bronchus beneath was divided by stapling, and lobectomy was completed. The postoperative course was uneventful and the patient was discharged on postoperative day 8. The present case is consistent with the SCARE criteria [2].

## 3 Discussion

We performed thoracoscopic left lower lobectomy in a lung cancer patient with AP. The number of case reports on this variation has been increasing in recent years, owing to the wider use of 3D-CECT angiography (Table 1). Pulmonary vascular variation is reported in 16.4 % of patients, of whom 47.8 % exist in the left lung [3]. An abnormal pulmonary vessel branch can potentially induce unexpected vessel injury, with life-threatening bleeding or accidental extra-lung resection. Careful surgery is required to dissect the branch that enters the preserved lobe. This is especially emphasized in video-assisted thoracoscopic lung resection, in which it is difficult to obtain a comprehensive picture of vessel anatomy. Preoperative 3D-CECT angiography reconstruction is helpful for identifying vessel anomalies and preventing such accidents. Hence, it is desirable to confirm vascular branching patterns routinely using this noninvasive investigation before anatomical lung resection, enabling surgeons to perform lung surgery more safely with fewer complications [4]. In patients with mediastinal branches of the pulmonary artery on the left side, vascular treatment should be managed by considering the possibility that the branches may enter either the lingular or basal segments [4].

**Table 1:** Reported cases of mediastinal basal pulmonary artery (AP).

Patient	Source		Variant AP branches	Age	Sex	Surgery	Surgical indication	Preoperative diagnosis	Interlobar fissure	size dominance of AP	Type of A4 + 5	Type of AP
1	1985	Banba	A^9^^+^^10^	55	M	OC-LUL	Lung cancer	+	Poor		Mediastinal	Independent
2	1994	Iwabuchi	A^5^^+^^8^	68	M	OC-LLL	Lung cancer	−	Poor		Combination	Common-trunk
3	1996	Sano	A^9^^+^^10^	70	M	OC-LLL	Lung cancer	+			Interlobar	Independent
4	2009	Moriyama	A^8^^+^^9^	75	M	VATS-LUL	Lung cancer	−			Interlobar	Independent
5	2010	Kataoka	A^5^^+^^8^^+^^9^^+^^10^	67	M	VATS-LUL	Lung cancer	−		+	Mediastinal	Common-trunk
6	2011	Sueda	A^8^	58	M	OC-LLL	Lung cancer	+			Interlobar	Independent
7	2012	Shibano	A^8^	56	M	OC-LUL	Lung cancer	−			Mediastinal	Independent
8	2012	Kaneda	A^9^	78	F	VATS-LLL	Lung cancer	−			Mediastinal	Independent
9	2012	Kozu	A^5^^+^^8b^	84	M	OC-LUL	Lung cancer	+			Combination	Common-trunk
10	2012	Matsumoto	A^5^^+^^8^^+^^9b^	78	M	VATS-LLL	Lung cancer	+		+	Combination	Common-trunk
11	2014	Kato	A^5b^^+^^8b^	79	F	OC-LUL	Lung cancer	−	Poor		Combination	Common-trunk
12	2014	Yajima	A^4^^+^^5^^+^^9^^+^^10^	74	M	VATS-LUL	Lung cancer	+		+	Combination	Common-trunk
13	2014	Kim	A^10^	52	M	No surgery	(Bacterial pneumonia)				Interlobar	Independent
14	2015	Kawai	A^8b^^+^^9b^^+^^10^	69	F	VATS-LLL	Lung cancer	+			Mediastinal	Independent
15	2015	Hong	A^3^^+^^4^^+^^7^^+^^8^^+^^9^	29	M	No surgery	(None)				Unknown	Common-trunk
16	2015	Hong	A^3^^+^^5^^+^^7^^+^^8^^+^^9^	66	M	No surgery	(Lung cancer)			+	Unknown	Common-trunk
17	2015	Hong	A^7^^+^^8^	72	M	No surgery	(Tuberculosis)				Unknown	Independent
18	2015	Hong	A^4^^+^^5^^+^^7^^+^^10^	64	M	No surgery	(Colon cancer)			+	Mediastinal	Common-trunk
19	2016	Sonoda	A^4^^+^^5^^+^^8^^+^^9^^+^^10^	73	M	VATS-LUL	Lung cancer	+			Mediastinal	Common-trunk
20	2016	Nagata	A^5^^+^^8^^+^^10^	Unknown	Unknown	OC-LUL	Lung cancer	+			Mediastinal	Common-trunk
21	2017	Sugiura	A^4^^+^^5^^+^^8^	70	M	OC-LUL	Lung cancer	+			Mediastinal	Common-trunk
22	2018	Mochinaga	A^8b^^+^^9b^^+^^10b^	60	M	VATS-S8 + 9 SEG	Lung cancer	+			Interlobar	Independent
23	2018	Yatsuyanagi	A^9^^+^^10^	65	M	VATS-LUL	Lung cancer	+			Mediastinal	Independent
24	2018	Nakano	A^4^^+^^5^^+^^8^^+^^9^^+^^10^	66	F	OC-LUL, S6 SEG	Lung cancer	+		+	Mediastinal	Common-trunk
25	2018	Mizukami	A^8^	76	F	VATS-LUL	Lung cancer	−			Mediastinal	Independent
26	2019	Uchida	A^5^^+^^8^^+^^9b^	65	M	VATS-LUL	Lung cancer	−	Poor		Combination	Common-trunk
27	2020	Iijima	A^4^^+^^5^^+^^8^^+^^9^	68	M	VATS-LUL	Meta. lung cancer	+	Poor	+	Mediastinal	Common-trunk
28	2022	Diong	A^5^^+^^9^	71	M	VATS-LUL	Lung cancer	+			Combination	Common-trunk
29	2022	Liu	A^8^	48	F	VATS-LUL	Lung cancer	+			Interlobar	Independent
30	2023	Agasthian	A^8^	69	M	VATS-LUL	Lung cancer	−	Poor		Combination	Independent
31	2023	Present	A^8a^^+^^9^	69	F	VATS-LLL	Lung cancer	+	Poor		Mediastinal	Independent

OC: open chest, VATS: video-assisted thoracoscopic surgery, LUL: left upper lobectomy, LLL: left lower lobectomy, SEG: segmentectomy, meta: metastatic, AP: Arteria Praebronchialis.

In the literature, we identified 30 patients with AP in the left lung other than the present case (Table 1). Among the 31 patients, the median age at diagnosis was 68.5 years (range: 29–84 years). Twenty-three cases (74 %) occurred in males, seven (23 %) in females, and one (3 %) was unknown, with definite male predilection [1]. Reports of this variation are all from Asian countries: 23 patients from Japan [5,6,[8], [9], [10], [11], [12], [13], [14],[16], [17], [18], [19]], five from Korea [1,15], and one each from China [3], Malaysia, and Singapore [7]. The racial predilection remains unclear. Twenty-six patients (84 %) underwent anatomical lung resection for left lung cancer, comprising 18 left upper lobectomies, seven left lower lobectomies, and one left S^8+9^ segmentectomy. The remaining five patients (16 %) had no surgery, one had pneumonia, one had lung cancer, one had colon cancer, and one had an unknown diagnosis. Among the 26 surgical patients, a preoperative diagnosis of AP was made in 17 patients (65 %) using the following modalities: 3D-CECT angiography in 12, two-dimensional (2D) CECT in 2, 2D-plain CT in 1, and angiography in 2. The remaining nine patients without preoperative diagnosis (35 %) underwent seven left upper lobectomies and two left lower lobectomies, and anomalous AP branches were recognized intraoperatively for the first time. Particularly in left upper lobectomies, incorrect division of the AP branch can occur by mistaking it for the mediastinal lingular artery, which could result in left pneumonectomy in the worst-case scenario. This occurred in two patients (Patients 4 and 30 in Table 1) during left upper lobectomy in the literature, and reconstruction of divided branches was performed to avoid pneumonectomy in both patients [6,7]. The diagnosis of AP among five patients without surgery was obtained by 2D-CECT in three patients and 3D-CECT angiography in 2. Furthermore, size dominance of the AP compared to the main pulmonary artery trunk was observed in 8 (26 %), and poor interlobar fissure development of the left lung was reported in six (21 %) of the studies. Consistent with the description by Hong et al. [1], no A^1^^+2^ or A^6^ branches originated from AP.

The branching pattern of AP is diverse [1] and is anatomically classified into the following four types (Table 2a): independent (15 patients, 48 %), common trunk with the lingular artery (13 patients, 42 %), common trunk with arteries other than the lingular artery (0 patients, 0 %), and common trunk with both the lingular artery and arteries other than the lingular artery (3 patients, 10 %). In the independent type, the AP branches independently from the main trunk of the pulmonary artery. In the common trunk type, the AP forms a common trunk with lingular artery branches, other than the lingular artery, or both. These results can be simplified as independent (15 patients, 48 %) and common trunk types (16 patients, 52 %), with one-half each.

**Table 2:** (a) Branching patterns of the lingular artery for the four groups of AP branching patterns (b) Branching patterns of AP for the four groups of lingular artery branching patterns.

(a)		
Branching pattern of mediastinal basal artery (patients, %)	Branching pattern of A^4^^+^^5^	Patients
(1) Independent (15 pts, 48 %)	(1) Interlobar	6
	(2) Mediastinal	7
	(3) Combination of both	1
	(4) Uncertain	1
(2) Common trunk with lingular a. (13 pts, 42%)	(1) Interlobar	0
	(2) Mediastinal	7
	(3) Combination of both	6
	(4) Uncertain	0
(3) Common trunk with other than lingular a. (0 pts, 0%)	(1) Interlobar	0
	(2) Mediastinal	0
	(3) Combination of both	0
	(4) Uncertain	0
(4) Common trunk with lingular a. & other than lingular a. (3 pts, 10 %)	(1) Interlobar	0
	(2) Mediastinal	0
	(3) Combination of both	1
	(4) Uncertain	2
pts, patients; a., artery.		


(b)		
Branching pattern of A^4^^+^^5^ (patients, %)	Branching pattern of mediastinal basal artery	Patients
(1) Interlobar (6 pts, 19%)	Independent	6
	Common trunk with lingular a.	0
	Common trunk with other than lingular a.	0
	Common trunk with lingular a. & other than lingular a.	0
(2) Mediastinal (14 pts, 45 %)	Independent	7
	Common trunk with lingular a.	7
	Common trunk with other than lingular a.	0
	Common trunk with lingular a. & other than lingular a.	0
(3) Combination of both (8 pts, 26 %)	Independent	1
	Common trunk with lingular a.	6
	Common trunk with other than lingular a.	0
	Common trunk with lingular a. & other than lingular a.	1
(4) Uncertain (3 pts, 10 %)	Independent	1
	Common trunk with lingular a.	0
	Common trunk with other than lingular a.	0
	Common trunk with lingular a. & other than lingular a.	2

pts: patients, a.: artery.

In contrast, among the 31 patients with AP, the branching pattern of the lingular pulmonary artery was categorized into the following four groups (Table 2b): purely interlobar (6 patients, 19 %), purely mediastinal (14 patients, 45 %), a combination of both (8 patients, 26 %), and uncertain (3 patients, 10 %). The mediastinal branching patterns of the lingular artery among AP cases accounted for 71 %, which was remarkably larger than that generally reported [5]. A summary of the lingular artery branching patterns among several literatures reported by He et al. [20] showed that mediastinal origin accounts for approximately 30 % and purely interlobar origin accounts for approximately 70 % in general. Based on the fact that AP branches that form the common-trunk with mediastinal lingular artery account for as many as 52 % of all reported AP cases (Table 2a and b), it is critical to understand the relationship between AP and the mediastinal lingular artery and discriminate them in surgery.

Table 2a shows the branching patterns of the lingular artery for the four groups of AP branching patterns. The independent type of AP (15 patients, 48 %) mainly consisted of a purely interlobar lingular artery in six patients and a purely mediastinal lingular artery in seven patients, equally divided in half. The common trunk with lingular artery type of AP (13 patients, 42 %) consisted of a purely mediastinal lingular artery in seven patients and a combination type of lingular artery (mediastinal and interlobar branches) in six patients, equally divided in half. No patients had a common trunk with other than lingular artery alone as a branching type of AP, and only three patients showed a common trunk with both lingular artery and other than lingular artery.

In contrast, Table 2b shows the branching patterns of AP in the four groups of lingular artery branching patterns. In the purely interlobar type of lingular artery (6 patients, 19 %), all 6 patients showed an independent branching pattern of AP. In the purely mediastinal type of lingular artery (14 patients, 45 %), half (7 patients) showed an independent branching pattern of AP, and the remaining half (7 patients) showed a common trunk with lingular artery pattern of AP, one half for each. In the combination type of the lingular artery (eight patients, 26 %), the common trunk with lingular artery pattern of AP accounted for almost all (six out of eight patients). These findings suggest that the embryonic origin of the AP intimately correlates with the mediastinal branching of the lingular artery.

## 4 Conclusion

A lung cancer patient with AP (A^8a+9^) underwent a thoracoscopic left lower lobectomy. Preoperative 3D-CECT angiography is effective for identifying this rare but potentially critical variation. Thirty-one AP cases in the literature demonstrated a branching pattern of AP as an independent or common trunk type, half of each. AP was also more frequently accompanied by a mediastinal lingular artery (71 %) than generally reported.

The following is the supplementary data related to this article.

Video 1 Operative findings.
Just before the separation of the most anterior portion of the interlobar fissure, the aberrant A^8a+9^ branch entering the lower lobe from the anteromedial aspect was ligated and divided to avoid injury. Video 1

## Consent

Written informed consent was obtained from the patient for publication of this case report and accompanying images. A copy of the written consent is available for review by the Editor-in-Chief of this journal upon request.

## Ethical approval

The publication of this case report has been approved by ethical committee (IRB) of Daiyu-kai General Hospital since August 19th, 2022 (#2022-012).

## Funding

This research did not receive any specific grants from funding agencies in the public, commercial, or not-for-profit sectors.

## Author contribution

The corresponding author performed the surgery and drafted the manuscript. Other authors participated in the surgery and revised and approved the manuscript.

## Guarantor

The corresponding author of this manuscript is the Guarantor, who accepts full responsibility for the work, had access to all the data, and controlled the decision to publish.

## Research registration number

1. Name of the registry: Research Registry.
2. Unique identifying number or registration ID: researchregistry9719.
3. Hyperlink to your specific registration (must be publicly accessible and will be checked): Research Registry (knack.com).

## Conflict of interest statement

The authors declare that they have no conflict of interest.
